# Using intersectional gender analysis to identify challenges in tuberculosis care at four health care facilities in Uganda

**DOI:** 10.1186/s40249-023-01171-3

**Published:** 2024-01-05

**Authors:** Winters Muttamba, Samson Omongot, Irene Najjingo, Roseline Nuwarinda, Esther Buregyeya, Mariam Otmani del Barrio, Rosemary Morgan, Bruce Kirenga, Sarah Ssali

**Affiliations:** 1 Makerere University Lung Institute, Makerere University, Kampala, Uganda; 2 Division of Infection and Global Health, School of Medicine, University of St Andrews, St. Andrews, UK; 3 Infectious Disease Institute, Makerere University, Kampala, Uganda; 4 School of Public Health, Makerere University, Kampala, Uganda; 5 UNICEF, UNDP/World Bank/WHO Special Programme for Research and Training in Tropical Diseases (TDR), World Health Organization, Geneva, Switzerland; 6 Johns Hopkins Bloomberg School of Public Health, Baltimore, MD USA; 7 School of Women and Gender Studies, Makerere University, Kampala, Uganda

**Keywords:** Tuberculosis, Gender, Intersectionality, Masculinity

## Abstract

**Background:**

Tuberculosis (TB) care could be considered as a continuum from symptom recognition, decision to seek care, diagnosis, treatment initiation and treatment completion, with care along the continuum influenced by several factors. Gender dimensions could influence TB care, and indeed, more men than women are diagnosed with TB each year. The study was done to identify social stratifiers that intersect with gender to influence TB care.

**Methods:**

A cross-sectional qualitative study was done at four health facilities in 3 districts in central Uganda between October 2020 and December 2020. Data was collected from patients seeking a diagnosis or on TB treatment through focus group discussions and key informant interviews. Key themes around gender guided by a gender and intersectionality lens were developed and thereafter thematic content analysis was done.

**Results:**

Women have increased vulnerability to TB due to bio mass exposure through roles like cooking. Women have increased access to health care services as they interface with the health care system frequently given their role as child bearers and child care givers. Men have a duty to provide for their families and this most often is prioritised over healthcare seeking, and together with belief that they are powerful beings leads to poor healthcare seeking habits and delays in healthcare seeking. Decisions on when and where to seek care were not straightforward for women, who most often rely on their husbands/partners to make decisions.

**Conclusions:**

Men and women experience challenges to TB care, and that these challenges are deeply rooted in roles assigned to them and further compounded by masculinity. These challenges need to be addressed through intersectional gender responsive interventions if TB control is to be improved.

**Graphical Abstract:**

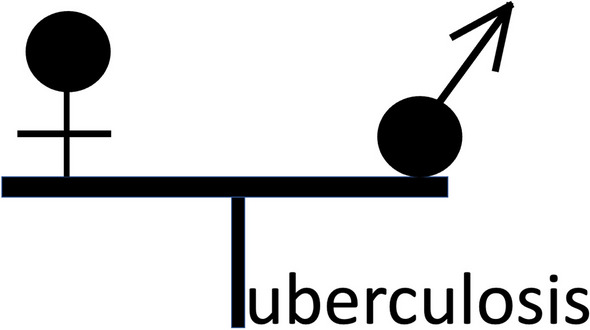

## Background

Tuberculosis (TB) is a major public health concern, one of the top ten causes of death worldwide and is a leading cause of death from a single infectious agent [1]. In 2022, up to 10.6 million people developed TB, with 1.3 million TB deaths recorded [2]. Uganda is one of the 30 high TB burden countries [3]. In Uganda, 91,000 people develop TB annually, with 15% of the TB cases occurring in children below 14 years [4].

TB care could be considered as a continuum from symptom recognition, to decision to seek care, to diagnosis, to treatment initiation and treatment completion, with care along the continuum influenced by several factors. A typical TB journey for most patients in Uganda is characterized by multiple health care provider visits before a diagnosis is made. This leads to delayed diagnosis and occasionally missed diagnosis [5]. Several factors could lead to delayed diagnosis, e.g. being a woman, previous HIV testing and poor knowledge on TB [6, 7].

Statistics show that more men than women are diagnosed with TB each year [8, 9], a reflection of how gender dimensions could influence TB care. While this could reflect the increased TB risk that men have for TB due to their social roles and involvement in activities that put them at an increased risk [9], it could also mean increased diagnosis in men and under diagnosis in women. The under diagnosis in women could be attributed to limited or lack of opportunities available to women for better TB care including social cultural disempowerment, stigma, different patterns of healthcare use, limited financial resources among others [10].

Differences within the same genders also exist such as differences in knowledge of TB, risk profile and burden of TB. For example, people in urban areas more likely to report self stigma [11]. Low knowledge and negative attitude to TB has been demonstrated among poor households, respondents with no schooling, young age groups (15–19 years) and rural areas [12].

It is important to appreciate the influence of gender and other intersecting factors in TB care, and intersectional gender analysis has been suggested as one way to do this. Gender influences the likelihood of TB disease, health care seeking and engaging with care and achievement of treatment success [13]. The concept of intersectional gender analysis has also been used in health programs through use of the intersectional gender score based on social covariables which allows for comparison of prevalence of individuals with a similar complex intersectional profile [14]. In TB, the use of sex aggregated data to inform TB programming has not been thoroughly done, and one of the reasons for this failure is limited nuanced analysis of the sex disaggregated data [14]. Intersectional gender analysis has been defined as “a process of analysing how gender power relations intersect with other social stratifiers to affect people’s lives, create differences in needs and experiences, and how policies, services and programmes can help to address these differences” [15]. It is built off the concept of intersectionality, which considers simultaneous interactions between different aspects of social identity, and allows for consideration of factors beyond favoured categories of analysis, for example, sex, gender, race, age, and class to consider simultaneous interactions between different aspects of social identity, as well as the impact of systems and processes of oppression and domination [15].

A study at Ugandan health care facilities was done to identify social stratifiers that intersect with gender to influence TB care (TB care seeking, diagnosis and treatment adherence), to understand how best to develop intersectional gender responsive interventions to control TB, as part of gender equitable TB programming.

## Methods

### Study design

This was a cross-sectional qualitative study nested within a bigger study (EXIT TB project). The EXIT TB project aims to implement and evaluate a TB screening package to increase TB case detection. Data was collected from patients presenting at study health facilities through focus group discussions and key informant interviews. Intersectional gender inequities were explored in relation to the study outcomes i.e., symptom recognition, health seeking, TB diagnosis, treatment, treatment initiation and adherence.

### Intersectional gender approach

The intersectional gender analysis toolkit [16] was used to guide development of research objectives and questions, and to incorporate gender analysis questions in the data collection tools and data analysis. Intersectional gender analysis influenced the design of data collection tools, selection of health facilities and participants, data analysis and manuscript writing. This approach enabled collection of data to inform the vulnerability, exposure, and TB experience, extent to which TB disease impacts individuals and the community, health care decision making process and adherence to TB treatment. The design of the study was informed by gender gaps in TB care, for example, it has been noted in literature that majority of missed TB cases are men [17]. Respondents were selected from three different districts (Buikwe, Kiboga and Nakaseke). Within the health facilities, the respondents were from the different service delivery points such as the outpatient department for participants with cough symptoms and from the TB unit for participants on treatment (initiating treatment, completed treatment or defaulted on treatment). When selecting the respondents and formation of focus groups, we ensured we did not create an environment that reinforced gender inequalities, thus separate groups for men and for women were created. The biggest worry with having focus groups consisting both men and women was it could affect responses from either sex and affect seamless and open discussion.

### Study setting

Interviews and discussions were held with participants from two urban health facilities in Buikwe and Kiboga districts, and two rural health facilities in Nakaseke district, all in central Uganda. The inclusion of both rural and urban health facilities was to help with exploring gendered differences between rural and urban areas through an intersectional gender lens. These health facilities are all public and offer health services free of charge, each has a TB diagnostic and treatment unit (DTU). They also act as referral centers for lower-level health facilities, and each serves approximately 400,000 people in its catchment. The annual total outpatient department (OPD) attendances are 91,947 for Kawolo hospital, 63,263 for Kiboga hospital, 27,479 for Nakaseke hospital and 27,169 for Kiwoko hospital [18]. The health facilities were selected given these were the same facilities where the parent project (EXIT TB) was being implemented.

### Sampling and study participants

The study team visited the four selected health facilities between October 2020 and December 2020 to introduce the study. Working with in charges of TB unit and OPD, participants for the focus group discussions (FGD) and key informant interviews (KII) were identified. The participants of interest included patients presenting at the OPD for cough symptoms and those attending the TB unit (initiating TB treatment, on TB treatment, completed treatment and defaulted on treatment).

To identify the participants, health facility registers were used to generate a line list of respondents at each level of the care cascade. Participants were selected through purposive sampling. A total of 17 FGDs were conducted (9 for males and 8 for females) and included 53 men and 54 women, with each FGD having 4–8 participants. A total of 26 key informant interviews were conducted (19 males and 7 females) were conducted.

Since the participants had also been accessing care at the health facility, the researchers did not contact them directly, but were contacted by the in charge of the TB unit under who they were or had received care. The in charge of the TB unit contacted them and requested them come to the health facility at the agreed time and date. At presentation to the health facility, the in charge of the TB unit briefed them about the study and those interested in participating were referred to the study team. The study team sought consent from the participants and conducted the interviews/discussions.

### Data collection

Participants were invited to take part in FGDs and KIIs at the health facilities. Separate FGDs were held for individuals at each level of the TB care cascade. Separate FGDs were held for men and women. Interviews/discussions were conducted within the health facilities in a private well aerated room to allow for physical distancing as this was during the COVID-19 pandemic. The discussions were facilitated by three researchers (IN, RN, SO) with training and experience in qualitative research. IN, RN identify as women and each have additional post graduate training in Public Health and Epidemiology, and SO identifies as a man, and has additional training in Public Health. The three were conversant in both English and the local language (Luganda). On average, the KIIs lasted 40 min while the FGDs lasted 1 h and were guided by an FGD and a KII guide that had previously been developed. The responses were recorded on two audio recorders. While the facilitator led the discussion, the note taker took field notes during the discussion. The FGDs and KIIs were stopped once saturation was achieved i.e. the point at which interviews produced little or no new useful information relative to the study objectives [19].

### Analysis

The audio recordings were transcribed verbatim by the two researchers (IN, RN) and later read separately by two other researchers (WM, SO) who familiarized themselves with the collected data. The researchers generated memos while going through the transcripts. First cycle of open coding was done manually, with codes developed using an inductive approach. The two researchers (WM, SO) later convened and reviewed the codes, refined/grouped them through consensus, and later themes that emerged were obtained and thereafter thematic content analysis was done.

### Ethics approval

The approval to conduct the study was obtained from Mulago hospital Ethics and Research Committee (Approval number MHREC-1339). The participants provided written informed consent.

## Results

Using gender as an entry point, factors (age, residence, marital status, employment status) that interact with gender and each other to affect TB care were identified. Several domains (risk of TB, TB knowledge, access to health information, health seeking behavior, masculinism, decision making power and autonomy) were developed, and in each of these domains, what men and women interface with to affect TB care was explored. The content synthesis and analysis are summarized in Table 1.

**Table 1:** Intersectional gender analysis matrix

Gender relations domains	Biological stratifiers					Disease domains^Ψ^			
	Age	Sex	Marital status	Residence	Employment	Risk of TB	Access to TB services	Health seeking behaviour	Access to resources
Gender roles	*	*	*	*	*	Roles assigned to women such as cooking increase their risk of TB	There are more opportunities for women to interface with health care facilities by virtue of their role as child care givers	Men delay health care seeking as they are more preoccupied with ensuring their families do not lack	Women encounter financial hardships to access health facilities, and also adhere on treatment
Masculinism	*	*	*	*	*			Men consider themselves powerful beings and thus present to health facilities late	
Decision making power and autonomy	*	*	*	*	*			Women rely on their husbands/partners to make decisions for them on when and where to seek care	

*Social stratifiers that intersect with gender to influence TB care

^Ψ^Illustrative examples of what emerged from the analysis showing how gender intersects with social stratifiers to influence important domains of TB care

### Increased TB risk due to gender roles

Men and women mentioned several TB risk factors, and identified who these TB risk factors affected the most. They linked some of the risk factors to the roles assigned to each one of them, and the opinions were influenced by where they live. For example, over half (68%) of the women in rural areas believed they were at an increased risk given their involvement in chores such as sweeping and cooking. On which age group was more affected, the respondents gave varying responses with some mentioning women aged 40 years and above.

*“Men do different work from women. Women do more of sweeping, cooking which makes it easy for them to get TB. So, I think TB is more in women than men.” FGD-Women completed treatment; Kiwoko hospital*.

*“40 years and above…I think its women.” KII-Men-lost to follow up, Kiwoko hospital.*

Men and women unanimously agreed that the economic status of an individual had a role to play in regard to TB risk. They mentioned low-income earners were more affected, with women among this group mentioned as being at greatest risk regardless of their residence.

*“Women have to depend on subsistence farming and it usually doesn’t yield much especially when the woman has no spouse.” FGD-Women initiated on TB treatment; Kawolo hospital.*

*“The disease we have is poverty. People look for those who have money. This ignorance mostly affects women because men have a good hand with money e.g., through fishing unlike women.” FGD-Women initiated on treatment; Nakaseke hospital.*

Men were mentioned as being more at risk of contracting TB compared to women, and were cited as less likely to seek care. The respondents attributed this to the roles the men play and engagement in risky behaviors such as smoking and alcohol consumption.

*“…mostly men considering the communal drinking at beer gatherings also known as "malwa…and it’s common in adult males.” FGD- Men on TB treatment, Nakaseke hospital.*

*“Mainly because of smoking and multiple sexual partners.” FGD-Females completed treatment; Nakaseke hospital.*

Respondents attributed the increased risk of infection in women to their roles in the household. The roles mentioned included cooking and sweeping.

*“Sweeping and cooking increases risk in women.” FGD-Female on TB treatment; Kiwoko hospital.*

*“Role in kitchen and the smoke effect makes us sick and hence come for care.” FGD-Female on treatment; Kiwoko hospital.*

### Knowledge on TB

Participants demonstrated mixed levels of understanding about the disease. From the discussions, some of what they knew about TB was from friends and the community as a whole. Both men and women described TB as a very dangerous disease with associated long-standing cough. The cough was described as being different from the usual cough and communities have several local terms to refer to TB. In more than 70% of the KIIs and FGDs, the most known symptom mentioned was cough, and participants mentioned the cough is different from the usual cough and takes long to heal. Most of the information the participants knew about TB was not in any way influenced by residence (rural or urban), however under a quarter of men linked some of the signs and symptoms to sexually transmitted diseases, with subsequent loss of sexual power.

*“TB is when you are infected with what we would call cough but it’s stronger than what we call the usual cough.” FGD-Men completed treatment; Kiboga hospital.*

*“I thought TB is when a person coughs but for my sake, my body started itching and it started from the private parts. I was told its syphilis and I started treating it.” FGD-Men on treatment Nakaseke hospital.*

### Access to health facilities

From the discussions, access to health facilities is influenced by financial resources. Both men and acknowledged health facilities as potential sources of information and medical care, and yet they are far from them. They mentioned they often have to incur transport costs to reach the health facilities. The women were more challenged than men, and of these, the unmarried were at a bigger disadvantage as they didn’t have partners to support them. The respondents cited several options were available for men to be able to visit the health facilities.

*“Women get more challenged; they don’t get enough support from their husbands.” KII-Women lost to follow up; Kawolo Hospital.*

*“A woman receives support from the husband to go to health facility which is not the case for men, men have to look for money first for transport.” FGD Completed Males-Kiwoko hospital.*

*“Several transport options available for men e.g., borrowing a bicycle….” FGD-Women on treatment; Kiwoko hospital.*

Respondents mentioned frequent visits to the health facilities make it hard for them to maintain their work schedules, and this further affected their financial resources. As a result of the demands to regularly return to the health facilities, most of them lose their regular sources of income. This was reported by all men and women from urban areas.

*“I became so weak that I couldn’t work, I resigned.” FGD-Woman on treatment; Kiboga hospital.*

*“Missing work because of treatment may lead to losing work.” KII- Man; Kawolo Hospital.*

The financial challenges in addition to affecting health care seeking also led to challenges with adherence to treatment, and influenced where participants sought care. From the discussions, the poor mainly go to public health facilities, with majority of those visiting the public health facilities being women. This was more mentioned by more than three quarters of the respondents in the rural areas, and this was influenced by finances.

*“The poor go to public hospitals, more so women.” FGD-Women on treatment; Kiwoko hospital.*

*“I don’t have the money. So, you can decide to go to a government health facility where they will not ask for money like in a big private hospital. At the health facility they will ask for a book or 10,000 Ugandan shillings and when they fail, they refer you to a big health facility.” KII-Woman, lost to follow up, Kiwoko hospital.*

### Gender roles and health seeking behavior

In regard to roles and practices assigned to men and women and their influence on health care seeking, respondents gave several opinions with more than half of the respondents alluding to the roles they are expected to play in the family. They mentioned several opportunities are available for women given their roles as women, e.g. their role in child bearing ensures women have to visit health facilities severally, e.g. for antenatal visits, post-natal visits and immunization of children. All the men agreed both in rural and urban areas agreed women’s role in child bearing puts them at an advantage.

*“Women come for antenatal so it’s easy to get help,” FGD- Men on treatment; Kiwoko hospital.*

*“Women frequent health facilities more than men, unless no money.” FGD – Men initiated on treatment; Kiboga hospital.*

*“Women frequent the health facilities more than we the men. They have more diseases than us. She might be already at the health facility and also tells the nurse that she has cough. Since they come for antenatal it’s easy for her to come when she feels not ok but we men find it hard.” FGD-Men Kiwoko hospital.*

Men and women described the contribution of the role of women as child care givers in TB control in different ways. Whereas the women mentioned the need to stay alive and take care of their children motivates them to seek care, men thought this role could affect women’s health care seeking as most times they are preoccupied with taking care of the children and find no time to visit health facilities.

*“Women mostly come, because they want to live and take care of children.” KII-Woman Completed treatment, Kawolo hospital.*

“…*they usually think about their children they say in case I die who will raise my children and for the men may not care about it so much.” FGD-Female initiated on TB treatment; Kiboga hospital.*

*“It is very possible children take women's time so much.” FGD-Men completed treatment, Kiwoko hospital.*

The respondents mentioned men were more likely to delay seeking care, and only seek care when the disease is advanced. It emerged that gender roles of men and the need to ensure their families are not lacking also affects healthcare seeking. This gender role assigned to men did not differ by income status or residence.

*“TB affects men worse because they have to work despite their sickness.” KII- Female lost to follow up; Kiwoko hospital.*

*“I first refused to come here because I had to work for money to feed children.” FGD-Male on TB treatment; Kiwoko hospital.*

### Masculinism

Masculinist tendencies emerged from the discussions and interviews. These influenced health care seeking patterns and delayed health care seeking. More than half the respondents cited men as having poor health care seeking behaviors, and presented late to the health facilities. The poor health care seeking and late presentation in men were attributed to the fact that they felt they are powerful beings.

*“Men pretend to be strong and don’t go to hospitals.” FGD-Females completed treatment Kawolo hospital.*

*“Men say am the boss for my life.” KII-Female lost to follow up; Kiwoko hospital.*

*“It’s so true a man does not fall sick and we have that mentality and people think it’s true. So by the time they go to health facilities it’s too late.” FGD-Males; Kiwoko hospital*

*“But men just don't want to go. We don't think everytime everytime you don't feel well you should run to hospital. These men who are like girls are the ones who run to the hospital.” FGD-Males Kawolo Hospital.*

### Decision making power and autonomy

Decision making is critical in health care seeking and the power to make decisions on when and where to seek care could be made by the patient themselves or could depend on a different person. We explored to what extent women were able access healthcare without permission from their partners. The unmarried women were able to easily make decisions as opposed to the married ones that had to first get permission from their partners.

*“My spouse gives me permission.” FGD- Females on treatment; Nakaseke hospital*

*“Woman looks upon a man, my wife would wait for my decision.” KII-Male lost to follow up; Kiwoko hospital.*

The decision-making ability for some was influenced by the fact that they have to rely on their partners for financial support. It was mentioned that women who had enough finances even when married could make their own decisions.

*“If I had my money, I would still decide by myself.” KII-Female lost to follow up Kiwoko hospital.*

*“…then if its far and you need money for transport especially for the married women who don’t work and they just have to ask for money. The man might say I don’t have money which makes her miss or stop the treatment.” KII-Female Lost to follow up; Kiwoko hospital.*

## Discussion

This study conducted at four health facilities in Uganda identified gender factors that affect TB care using an intersectional analysis lens. The factors span the domains of: TB risk, TB knowledge, access to health information, health seeking behaviour, masculinism, decision making power and autonomy.

From the discussion, both men and women acknowledged they are at increased risk for TB and the roles each gender plays influence the TB risk profile. Women aged 40 years and above in rural areas were mentioned as being at greatest risk because of their roles in the household. Such roles included cooking and sweeping, and this is most likely linked to biomass exposure as studies have documented women get exposed to pollution while cooking using biomass fuel [20]. Studies have documented high concentrations of particulate matter 2.5 (PM_2.5_) and carbon monoxide (CO) during cooking [21]. Exposure to biomass increases risk of TB risk as has been reported in several studies [22–24], and given the increased role of women in cooking in most African settings, this is likely to be a big contributor. The respondents mentioned biomass exposure was more among the rural dwellers than the urban dwellers. This is supported by other studies that showed wood was the most frequent fuel used for cooking in rural areas [25]. The socio-economic status of women emerged as a risk factor with women belonging to the low-income status listed as being at greatest TB risk. Low economic status as revealed in this study has also been previously found to be associated with increased TB risk, with gender differences in economic roles and activities leading to different exposure to the bacilli [26, 27].

Access or lack of access to financial resources also affected the men and women and influenced TB care seeking. The financial constraints were more noted among females than males, with the married women having a better advantage over their unmarried counterparts as the former received financial support from their husbands/partners to access care. Females most often have to rely on their partners to support their health care seeing visits, and the financial dependence among females has previously been documented [28].

The study revealed challenges with decisions making for women, reinforced by lack of autonomy especially among the married women. Married women most often have to seek care from their husbands before they can visit the health care facilities, and this is more pronounced in scenarios where the women do not have financial independency. The lack of decision making has been documented in previous studies [28]. The limited or lack of employment for women and the predominant role of men in cushioning the household financial burden occasionally stands in the way of women’s decision making [29], and indeed, it was noted during the interviews and discussions that women who had enough finances even when married could make their own decisions without having to first get approval from their partners. A previous study has documented that women who report to be decision makers (whether main or joint decision makers) are more likely to be involved in work outside their household or report earning more than their husbands [30]. The health outcomes are better when women’s power is appreciated by the partner [30].

The socially constructed roles for men and women in the community also affected the health care seeking options and behaviours. Women by virtue of their role as child care givers had more opportunities to interface with the health care facilities. As a result of women and child services available that bring women to the health facilities, some have described the health facilities as women spaces [31]. Closely related to this was the call for women to stay alive so as to take care of their children, and thus motivating them to seek health care. It has been reported that women typically cushion up to 75% of childcare responsibilities [32]. The increased interaction between women and health care facilities has been documented in a population based study that reported more women than men had attended at least one health care visit in the preceding 60 days, with majority of the women being pregnant or carers of children aged under 2 years [33].

Masculine ideas that portray men as being strong beings emerged during the study, and these were highlighted as being responsible for the men’s delayed health care seeking. Previous studies have documented men are more likely to delay health care seeking following a cough symptom [34]. Masculinist tendencies that view men as strong and perceive health care seeking as a sign of weakness have been documented in other studies [35–37]. Despite women presenting early to the health facilities, it has been documented in several that they wait longer periods than men before accessing a diagnosis [38, 39]. The reasons for this are not well elucidated but some studies point to organisational and policy factors [38]. It is possible men get diagnosed faster when they present to the health care facilities as most often, they present late when the disease is advanced and most likely able to turn positive. This is supported by a study that demonstrated shorter healthcare delay is associated with a positive sputum smear [39].

This study had several limitations. We recruited participants who were either being managed or had been managed for TB at the study facilities. This may have led to selection bias, given these participants might have opinions different from the participants that stay in their communities who don’t make any attempt to getting to the health facilities. To limit this, we asked the participants to describe their treatment journeys right from the onset of symptoms. This might be the same journey everyone goes through before they get treatment.

## Conclusions

Despite the use of sex-disaggregated data in TB programs, lack of nuanced analysis of this data might be one of the reasons for slow progress in improvement of TB control indicators. The results from this study are thus an entry point into more robust and nuanced analysis of sex-disaggregated program data. This study has been able to document that different groups of men and women experience challenges to TB care, and that these challenges are compounded by other factors that interact with various gender dimensions. It is important that tailored interventions are designed and implemented with this approach in mind to ensure they respond to the differences in needs and experiences by different population subgroups if interventions are to produce the anticipated benefits.

## Data Availability

Available from the corresponding author on reasonable request.

## Abbreviations

TB: Tuberculosis
HIV: Human immunodeficiency virus
DTU: Diagnostic and treatment unit
FGD: Focus group discussion
KII: Key informant interviews
